# Long-Term Assessment of Intraocular Lens Stability, Tilt and Decentration Between Four-Point Scleral Fixation and Yamane Techniques

**DOI:** 10.3390/jcm15082967

**Published:** 2026-04-14

**Authors:** Natalia Blagun, Karolina Krix-Jachym, Marek Rekas

**Affiliations:** Ophthalmology Department, Military Institute of Medicine—National Research Institute, Szaserów Street 128, 04-141 Warsaw, Poland

**Keywords:** IOL tilt, IOL decentration, four-point scleral fixation, Yamane technique, AS-OCT

## Abstract

**Background**: To compare tilt and decentration results of two scleral fixation intraocular lens (IOL) methods of four-point scleral fixation (Akreos AO60) and the Yamane technique (AcrySof MA60AC). **Methods**: Two groups were compared in terms of IOL decentration and tilt at day 30 and 2 years after surgery. Correlations between IOL tilt and decentration and previous pars plana vitrectomy (PPV), axial length (AL), refractive error (RE), astigmatism, and spherical equivalent (SE) were also analyzed. **Results**: This study included 50 eyes from 47 patients: four-point fixation in 25 eyes (group 1) and Yamane technique in 25 eyes (group 2). The mean horizontal tilt was 1.66° ± 1.45° in group 1 and 5.06° ± 4.65° in group 2. The horizontal tilt value was significantly higher (*p* < 0.05) in group 2. The mean vertical tilt for group 1 and group 2 at two year’s observation was 1.74° ± 2.16° and 3.09° ± 2.79° respectively (*p* = 0.02). The mean horizontal IOL decentration was 0.35 ± 0.32 mm in group 1 and 0.34 ± 0.26 mm in group 2 (*p* > 0.05). The mean vertical IOL decentration in group 1 was 0.34 ± 0.33 mm and in group 2 it was 0.27 ± 0.20 mm respectively (*p* = 0.45). No statistically significant changes in tilt and decentration time were demonstrated in both groups (*p* > 0.05). There was no statistically significant correlation between IOL tilt and decentration and previous PPV in both groups. **Conclusions**: Both techniques provide good centration and stability IOL in aphakic eyes in the absence of capsular support.

## 1. Introduction

Proper positioning of the intraocular lens (IOL) is the basis for optimal visual function after surgery. One of the complications after phacoemulsification surgery or scleral fixation techniques is that the IOL may be tilted after surgery. Ashena et al. in their study report that IOL tilt has been noticed more frequently with scleral fixated IOLs compared with in-the-bag IOLs [1]. The increased extent of IOL tilt and decentration can have a negative effect on optical performance and cause oblique astigmatism, causing wavefront aberrations and patient’s satisfaction [2,3].

In eyes without adequate capsular support, obtaining stable centration and minimizing tilt is particularly challenging and depends largely of the fixation technique used. These problems have been addressed by the development of different scleral fixation techniques, which vary in the geometry of the attachment, the number of points of fixation, haptic externalization and stabilization, the use of sutured or sutureless techniques, scleral tunnel design, and the characteristics of the implanted IOL [4,5,6]. The fact that numerous techniques have been proposed over the years reflects the lack of a universally accepted standard and highlights the need for further comparative studies, concentrating not only on safety and visual outcomes but also on the long-term stability of lens position.

Among contemporary techniques, four-point scleral fixation and sutureless fixation using the Yamane technique are widely used approaches in the treatment of aphakia. Although both methods aim to ensure stable IOL positioning and satisfactory visual outcomes, differences in the mechanics of fixation may influence the postoperative positioning of the lens. Therefore, a direct comparison of these techniques is of significant clinical importance for a better understanding of their impact on IOL positioning and its long-term stability.

Modern optical measurement techniques such as higher solution anterior segment optical coherence tomography (AS-OCT) are capable of measuring the axial position (front and back apex position), decentration and tilt of the crystalline lens/IOL before/after cataract surgery [7]. This is a non-invasive and non-contact technique to measure the tilt and decentration of the IOLs. Wang et al. reported a high inter-operator and inter-session reproducibility with this convenient and high-resolution imaging technique, using a 3D reconstruction method for their calculations [8].

The purpose of our study was to compare the tilt and decentration of two methods of scleral fixation IOL: four-point fixation of the Akreos AO60 IOL (Bausch & Lomb) using polypropylene sutures and two-point fixation of AcrySof MA60AC IOL (Alcon) using the Yamane technique. Moreover, our aim was to assess their effect on postoperative refractive changes. We also analyzed correlations between tilt and decentration and biometric parameters and previous pars plana vitrectomy (PPV).

## 2. Materials and Methods

Our study was prospective and was conducted at the Military Institute of Medicine—National Research Institute in Warsaw between 2021 and 2025. The study was conducted in accordance with the tenets of the Declaration of Helsinki and was approved by the Bioethics Committee of the Military Institute of Medicine (NR 11/WIM/2021). Informed consent to participate in the study was obtained from all subjects. Fifty eyes from 47 patients with aphakia without capsular support were included in the study. Patients were enrolled consecutively as they presented and met the inclusion criteria prior to randomization. Patients were assigned to two study groups during their first visit using computer-generated randomization lists based on a random sorting algorithm (maximum allowable deviation of 10%, two iterations). The 4-point fixation technique was used for the first group. The 2-point fixation using the Yamane technique was used for the second group.

All the patients received ophthalmic examinations before surgery. Exclusion criteria for this study included corneal diseases (corneal dystrophy, corneal haze, or scarring, history of corneal transplantation), astigmatism of more than 2.0 D (diopters), macular diseases (age-related macular degeneration, diabetic maculopathy), other retinal diseases (retinal detachment, retinal vein occlusion), and other ocular diseases that may affect visual acuity (traumatic optic neuropathy).

**Figure 1:**
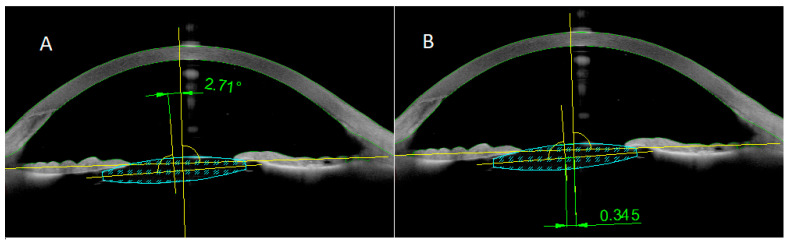
Anterior segment optical coherence tomography (AS-OCT) images. (**A**) tilt; (**B**) decentration.

Preoperative biometry was performed using the Zeiss IOL master 700 (Carl Zeiss Meditec, Jena, Germany), and the theoretical Sanders–Retzlaff–Kraff (SRK/T) formula was used to calculate lens power. Lens decentration and tilt were assessed using anterior segment AS-OCT (Anterion, Heidelberg Engineering, Germany). Li et al. reported accurate measurement of the IOL tilt and decentration using this method [9]. The IOL tilt is defined as the angle between the intraocular lens axis and the reference line (Figure 1A). The distance between the midpoint of the reference line and the IOL center was measured as IOL decentration (Figure 1B). Tilt angle and decentration were measured in all patients at 1 month and 2 years after surgery.

BCVA was collected in decimal visual acuity values and converted to logMAR for statistical analysis. Postoperative total refractive error (RE) was calculated as the difference between expected and actual refractive error after surgery.

All statistical analyses were performed using StatSoft, Inc. STATISTICA version 13.0. Quantitative variables were described using mean, standard deviation, median, minimum and maximum values (range), and 95% confidence intervals (95% CI), while qualitative variables were presented as counts and percentages.

The normality of distribution for continuous variables was assessed using the Shapiro–Wilk test. Variables with a normal distribution were analyzed using parametric tests (Student’s *t*-test), whereas non-normally distributed variables were evaluated using non-parametric tests (Mann–Whitney U-test). For paired data, Student’s *t*-test or Wilcoxon signed-rank test was applied, as appropriate.

Associations between categorical variables were analyzed using the Chi-square test of independence. Correlations between variables were assessed using Pearson’s or Spearman’s correlation coefficients, depending on data distribution.

All statistical tests were two-sided, and a *p*-value < 0.05 was considered statistically significant.

### Surgical Technique

All surgeries were performed by a single surgeon (MR). A hydrophilic acrylic one-piece AcrySof AO60 IOL (Bausch & Lomb, Rochester, NY, USA) was used for four-point fixation in group 1. This IOL was fixated with a 6-0 polypropylene suture. For group 2, a three-piece AcrySof MA60AC IOL (Alcon, Fort Worth, TX, USA) was used for sutureless fixation using the Yamane technique.

Group 1—Four-point scleral fixation of Akreos AO60 IOL

After retrobulbar anesthesia, four intrascleral tunnels were marked 2 mm from the limbus and spaced 6 mm apart. Corneal incisions were made, the anterior chamber was filled with a pupil-dilating solution and viscoelastic, and core vitrectomy was performed if needed. An IOL was implanted into the anterior chamber using an injector. A 6-0 polypropylene suture was introduced through corneal incisions, then guided through a 30G needle via the haptic openings and externalized through sclerotomies. The procedure was repeated for the second haptic. The IOL was centered by adjusting the suture tension, and the ends were cauterized to create flanges, then fixed subconjunctivally. Finally, the corneal incisions were sealed and an antibiotic was injected into the anterior chamber.

Group 2—Sutureless scleral fixation of AcrySof MA60AC IOL with Yamane technique

The surgery was performed under retrobulbar anesthesia. Intrascleral tunnel sites were marked 2 mm from the corneal limbus, positioned 180° apart to prevent lens tilt. Corneal incisions were made, and pupil-dilating solution along with viscoelastic was injected into the anterior chamber. Anterior vitrectomy was performed if vitreous was present. A three-piece IOL was implanted into the anterior chamber. A 30G needle was used to create the first sclerotomy 2 mm from the limbus, and one haptic was threaded through the needle and externalized. The same was done for the second haptic on the opposite side. The haptic tips were cauterized and placed into the scleral tunnels and covered with conjunctiva. After sealing the corneal incisions, an antibiotic was injected into the anterior chamber.

These surgical techniques were described in more details previously [10].

## 3. Results

The study included a total of 50 eyes from 47 patients with aphakia, comprising 23 females and 24 males. The mean age was 67.8 ± 13.5 years in group 1 and was 75.0 ± 10.3 years in group 2 (*p* > 0.05). As for the axial length, it was similar in both groups. The prevalence of myopia was equivalent in both groups (12%). Previous PPV was performed in 28% of patients in group 1 and 20% in group 2. No statistically significant differences were found between study groups regarding demographics (*p* > 0.05). These data are presented in detail in Table 1.

Table 2 presents the results of measurements of the IOL tilt and decentration at the horizontal and vertical meridians in both groups at one month and two years after surgery. The mean vertical and horizontal decentration were similar in both groups and showed no statistically significant differences at both 1 month and 2 years after surgery (*p* > 0.05). Additionally, there was no statistically significant change in horizontal or vertical IOL decentration in group 1 (*p* > 0.05) and group 2 (*p* > 0.05) at the two-years follow-up compared to one month follow-up. We found no differences between the groups regarding vertical tilt on day 30 follow-up (*p* > 0.05). But the mean vertical tilt was significantly high in group 2 at two-years follow-up (*p* = 0.02). However, the angle of horizontal tilt was significantly lower in four-point fixation group compared to the Yamane technique group (*p* < 0.05). We found no increase in the horizontal or vertical tilt in either group over the course of the observation period. These results indicate good long-term stability of the IOL in both groups. Although statistically significant differences in tilt were observed, their impact on visual function appeared to be limited, as evidenced by the absence of significant differences in visual acuity between the groups.

**Figure 2:**
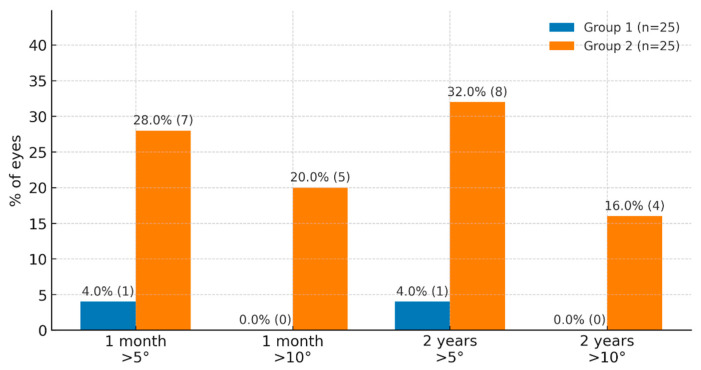
Comparative characteristics of group 1 and group 2 in terms of horizontal tilt (visit 1 month, visit 2 years).

Horizontal tilt values greater than 5° were observed in 4.0% of cases in group 1 and in 28.0% of cases in group 2 at the one-month postoperative visit (*p* = 0.02). No horizontal tilt values greater than 10° were observed in group 1 at the one-month follow-up. However, 20.0% of such cases were observed in group 2 at the same follow-up (*p* = 0.02). Horizontal tilt values greater than 5° were observed in 4.0% of cases in the four-point fixation group, and in 32.0% of cases in the Yamane technique group at two years follow-up. The difference was statistically significantly higher in group 2 (*p* = 0.01). In the group 1, no horizontal tilt values greater than 10° were observed over the 2-year observation period. However, 16.0% of such cases were observed in group 2 at the same follow-up (*p* = 0.04). However, despite this difference, no significant differences in BCVA were found between the groups, suggesting limited direct clinical significance. Therefore, the clinical significance of the observed differences in tilt should be interpreted with caution. Figure 2 shows the percentage of horizontal tilt greater than 5° and 10° in both groups after one month and two years of follow-up.

**Figure 3:**
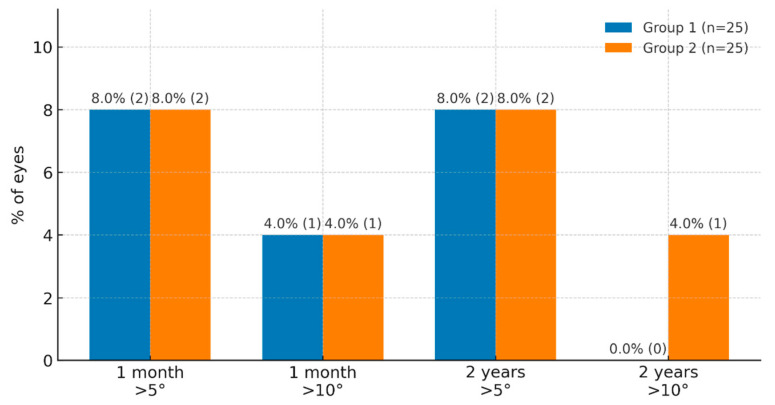
Comparative characteristics of group 1 and group 2 in terms of vertical tilt (visit 1 month, visit 2 years).

Figure 3 presents the percentage of eyes with vertical IOL tilt greater than 5° and 10° in both groups at 1 month and 2 years postoperatively. At day 30, vertical tilt > 5° was observed in 2 eyes (8.0%) in each group (*p* = 1.00), while tilt > 10° occurred in 4.0% of cases in both groups (*p* > 0.05).

At the 2-year follow-up, vertical tilt > 5° remained present in 8.0% of eyes in both groups (*p* = 1.00). No cases of vertical tilt > 10° were observed in group 1, whereas 4.0% of such cases were noted in group 2 (*p* = 0.31). In summary, the distribution of tilt values remained stable over time in both groups and showed no tendency to increase.

We found no cases of IOL decentration values above 1.0 mm in both groups.

Mean postoperative BCVA for group 1 and group 2 was 0.10 ± 0.15 logMAR and 0.09 ± 0.17 logMAR respectively (*p* > 0.05). There were no statistically significant differences between groups in terms of BCVA at any follow-up period (*p* > 0.05). There was no correlation between IOL tilt, decentration and BCVA in both groups.

Table 3 shows the postoperative results of visual acuity and refractive error at the two years after surgery for both groups. Postoperative spherical equivalent (SE) refractive error and total postoperative RE were significantly higher in group 2. The increase in horizontal tilt was correlated with the values of cylinder (correlation coefficient R = 0.40, *p* = 0.04) in group 2. In group 2, the increase in horizontal tilt was associated with the increase in total RE (R = 0.41, *p* = 0.04). There were no statistically significant correlations between decentration IOL and RE, cylinder or spherical equivalent (SE) in both groups. This suggests that tilt may affect refraction test results, but these correlations did not result in differences in visual acuity. These results may have been influenced by the relatively small number of patients in the study.

There was no statistically significant correlation between IOL tilt, decentration and AL in both groups. The IOL tilt and decentration results were similar in both groups. Table 4 shows results of IOL tilt, decentration, and previous vitrectomy in both groups at the two-years follow-up. There were no statistically significant differences between study groups related to previous PPV (*p* > 0.05). Equally, no correlation was found between IOL tilt, decentration and previous PPV in either group. However, the relatively small number of patients with previous PPV compared to those without PPV may have limited the statistical power to detect potential differences; therefore, these findings should be interpreted with caution.

Late complications included IOL displacement, suture extrusion, haptic extrusion and pseudophakic reverse pupillary block (PRPB). IOL displacement was observed in one case (4%) in group 2, and required lens repositioning. Suture extrusion was noted in one case (4%) in group 1 nine months after surgery. Haptic extrusions were noted in two cases (8%) in group 2 after surgery (*p* > 0.05). Conjunctival plastic surgery was performed and no re-extrusion was observed. PRPB was diagnosed in two cases (8%) several months after surgery in group 2, and peripheral laser iridotomy (LPI) was performed.

## 4. Discussion

One of the complications following phacoemulsification surgery or scleral fixation techniques is incorrect IOL position, which can result into visual dysfunction. In our study, we described the results of IOL tilt and decentration of these two methods and assessed their effect on refractive outcomes. Additionally, we examined the correlation between previous PPV, AL and IOL tilt and decentration in both groups.

The causes of IOL tilt and decentration can be preoperative, intraoperative and postoperative. Preoperative causes, contributing to the IOL decentration and tilt, can include AL and previous PPV.

The literature suggests that the IOL tilt is significantly associated with a shorter axial length—the IOL tilt decreases by 0.228° for every 1 mm increase in AL after cataract surgery [11]. Furthermore, a greater IOL decentration is correlated with a longer AL. This means that the position of IOL is more likely to tilt in patients with high myopia decenter postoperatively and with high hyperopia or microphthalmia [12]. These studies report the relationship between the AL after cataract surgery and IOL implantation into the capsular bag. In our study, mean AL was similar in both groups and was 24.23 ± 2.0 mm in group 1 and 23.82 ± 1.2mm in group 2 (*p* > 0.05). We found no statistically significant correlation between IOL tilt, decentration and AL in either group. Such results might be caused by an insufficient number of patients with either very short or very long AL. Absence of capsular bag support for the IOL could be another factor affecting IOL tilt and decentration. Lens capsular bags loosen with elongation of ALs, and thus, IOLs are likely to shift in larger capsular bags of long AL eyes [13].

IOL scleral fixation is frequently combined with vitrectomy. Tan et al. report that patients who underwent phacoemulsification after PPV had greater IOL tilt and decentration than the non-PPV group. They found that the mean IOL tilt and decentration were greater in the PPV group (5.36 ± 2.50 degrees and 0.27 ± 0.17 mm, respectively) than in the non-PPV group (4.54 ± 1.46 degrees, *p* = 0.005; 0.19 ± 0.12mm, *p* < 0.001, respectively) at 3 months follow-up. Duration of silicon oil (SO) tamponade was positively correlated with IOL tilt (*p* = 0.014) and decentration (*p* < 0.001) [14]. Chen et al. report in their multivariate analysis of IOL tilt that a larger degree IOL tilt was positively associated with previous PPV surgery (*p* = 0.014) and negatively associated with AL (*p* < 0.001) [11]. They additionally noted in the same study that patients with an IOL tilt greater than 10 degrees all had previously undergone PPV surgery. These findings indicate that previous PPV surgery and shorter AL are strongly correlated with greater IOL tilt. Both of those studies show the effect of previous PPV on IOL tilt and decentration for patients after phacoemulsification with IOL capsular bag implantation. Our study examined the effect of previous PPV on IOL tilt and decentration after scleral fixation. The mean results of IOL tilt and decentration for patients with and without previous vitrectomy were similar in both groups in our study and were not statistically different. We found no correlation between previous vitrectomy and IOL tilt or decentration. This means that both techniques can be used for patients, regardless of previous vitrectomy.

IOL decentration was similar to what has been previously described in the literature. Sül et al. reported that the decentration of the sutureless Yamane technique was 0.28 ± 0.09 mm at the horizontal meridian and 0.33 ± 0.12 mm at the vertical meridian [15]. In Schranz’s et al. study, the mean IOL decentration was 0.57 ± 0.37 mm in patients, where Yamane technique was used [16]. In the study by Wu et al., the average IOL decentration was 0.23 ± 0.12 mm horizontally and 0.18 ± 0.13 mm vertically in patients who used the Akreos AO60 4-point IOL fixation technique [17].

Results of IOL tilt in both groups are consistent with earlier studies that reported IOL tilt after conventional phacoemulsification surgery [15,18,19]. Yamane et al. reported that the mean IOL tilt angle was 2.5° ± 2.0° on the horizontal axis and 2.2° ± 1.8° on the vertical axis in the non-sutured intrascleral fixation technique [20]. Similar results were reported by Kumar et al. study, who underwent sutureless intrascleral fixation, the three-piece IOL being fixated with fibrin glue [21]. In Kemer’s et al. study, three-piece intraocular lens (Acrysof MA60AC, Alcon) was fixated using scleral Z-suture technique. They found that the mean IOL tilt angle was 3.8° ± 4.09°on the horizontal axis and 2.83° ± 4.03° on the vertical axis [22]. However, Schranz et al. in their study found higher IOL tilt (the mean IOL tilt was 7.67° ± 3.7°) in the group of patients, where the Yamane technique was used [16]. In our study, the mean postoperative horizontal tilt angle was 5.06° ± 4.65°, which was significantly higher than the vertical tilt of 3.09° ± 2.79° in the Yamane technique group. Wang et al. report in their study that the mean IOL tilt was 3.74° ± 1.31° through double-suture four-point flange intrascleral fixation [23]. Similar results were obtained in our study for patients in group 1. However, the mean angle of horizontal tilt was significantly lower in the four-point fixation group than the Yamane technique group.

The horizontal tilt angle of >5° was observed in 4.0% cases in the four-point fixation group and in 32.0% cases in the Yamane technique group (*p* = 0.01). Horizontal tilt angle > 10° was not observed at two-years follow-up in the first group but was found in 16.0% of cases in the second group at two-years follow-up. These values are similar to those reported in previous studies by Kemer’s et al. after scleral fixation [22].

Refractive outcomes after the procedure were similar in both groups in our study. However, the refractive error was higher in patients who underwent surgery using the Yamane technique. We found that an increase in horizontal tilt was associated with an increase in refractive error in group 2. Results were consistent with previous studies that also reported the effect of IOL tilt on higher-order aberrations and refractive error [24,25]. Increase in horizontal IOL tilt in group 2 might be related to the fact that different types of IOLs for scleral fixation were used in our study. The haptic design was most likely responsible for the lower IOL tilt when using the Akreos AO60 lens. Schranz et al. reported in their study that scleral fixation of a three-piece IOL in the Yamane technique induces torque and stress on the haptic–optic junctions, subsequently leading to IOL tilt. Another reason of IOL tilt is that the haptics are passed through an intrascleral tunnel whereby the length and angle of the tunnel in the eye are prone to being asymmetrical despite the external placement of the 30G needle 180° apart [16]. To reduce IOL tilt in patients, where the Yamane technique was used, Kurimori et al. proposed shortening the length of the haptics [26]. Later, Lin et al. analyzed the results of 19 patients who underwent surgery with untrimmed haptics and 20 patients with trimmed haptics, where the Yamane technique was used. They concluded in their study that the position of the IOL may change with time, but trimming the haptic to an optimum length tends to provide greater IOL stability [27]. In our study, the haptics of three-piece IOL were not trimmed, which may have affected the greater horizontal tilt angle.

New advances in scleral fixation techniques focus on improving the stability of IOL and reducing their tilt and decentration. The innovative needle-guided single-suture technique, described by De Luca et al., represents an alternative approach aimed at simplifying the procedure whilst maintaining adequate IOL centration through modifications to the fixation geometry and haptic control. Although this study focused primarily on surgical feasibility and safety, no cases of tilt or decentration >0.3 (the mean tilt angle was 2.1 ± 0.7°) were observed [28]. In this context, our findings regarding IOL tilt and decentration further confirm the significant role of fixation technique in postoperative IOL stability. Although different techniques may produce comparable visual outcomes, slight differences in IOL position—such as the greater tilt observed with some methods—may relate to the scleral fixation technique. Therefore, new approaches, such as the needle technique, need further comparative studies, including detailed assessment of IOL tilt and decentration using objective visualization methods.

The study data shows good visual results and long-term stability for both proposed methods. Our study demonstrated same low IOL tilt and decentration, as reported by other authors. IOL tilt results were lower when using four-point scleral fixation with the Akreos AO60 IOL, allowing for more predictable refractive outcomes after surgery.

The advantage of our study is a relatively large number of patients and a follow-up time of two years. An additional advantage was that we assessed the effects of previous vitrectomy and axial length on IOL tilt and decentration results.

There are also some limitations of our study. One of them is the non-evaluation of higher-order aberrations in relation to IOL tilt and decentration. Secondly, the sample size was relatively small and only a small number of bilateral cases were included, which may have limited the statistical power of the analyses. For further research, larger samples with more cases of high myopia and hyperopia are needed to examine their effect on lens position.

## 5. Conclusions

Both analyzed techniques provide long-term stability and good centration of the IOL in aphakic eyes in the absence of capsular support. In our study, we compared IOL tilt and decentration for the two most-used scleral fixation techniques. In summary, IOL tilt and decentration are commonly found in eyes after scleral fixation. The IOL tilt for four-point fixation is lower compared to the Yamane technique, which had an impact on the refractive error. The four-point fixation method allows for more predictable refractive outcomes after surgery. No significant association was detected in this sample of previous vitrectomy on IOL tilt and decentration, allowing both methods to be used in patients after vitrectomy. The study of different scleral fixation methods of intraocular lenses helps surgeons to plan the procedure and choose the surgical technique that provides the best results for the patient.

## Figures and Tables

**Table 1:** Baseline patient demographics and ocular history.

Characteristics	Group 1 (Akreos AO60)	Group 2 (MA60AC)	*p*-Value
Total patients (no.)	23	24	
Total eyes (no.)	25	25	*p* > 0.05 ^3^
OD (no.,%)	13 (52.0%)	10 (40.0%)	
OS (no.,%)	12 (48.0%)	15 (60.0%)	
Gender			
Female (no.,%)	10 (40%)	15 (60%)	*p* > 0.05 ^3^
Male (no.,%)	15 (60%)	10 (40%)	
Age			
Mean ± SD (years old)	67.8 ± 13.5	75.0 ± 10.3	*p* > 0.05 ^1^
Range (years)	38.0–86.0	53.0–92.0	
Follow-up time			
Mean ± SD (days)	721.5 ± 42.1	744.4 ± 71.3	*p* > 0.05 ^2^
AL (mm, mean ± SD)	24.23 ± 2.0	23.82 ± 1.2	*p* > 0.05 ^2^
Ocular History (%)			*p* > 0.05 ^2^
PPV	7 (28%)	5 (20%)	
Myopia	3 (12%)	3 (12%)	

Abbreviations: no.—number; OD—right eye; OS—left eye; SD—standard deviation; AL—axial length; PPV—pars plana vitrectomy; ^1^ t-Student; ^2^ U Mann–Whitney; ^3^ Chi-square.

**Table 2:** The angle of the IOL tilt and decentration measurements at the vertical and horizontal meridians in the four-point scleral fixation and Yamane technique at 30 days and two years after surgery.

IOL Position	Four-Point Scleral Fixation (Akreos AO60)	Yamane Technique (MA60AC)	*p*-Value
Horizontal meridian 1 month			
Tilt (degree, Mean ± SD)	1.54° ± 1.68°	4.98° ± 0.74°	*p* < 0.05 ^1^
Decentration (mm, Mean ± SD)	0.37 ± 0.25	0.37 ± 0.30	*p* = 0.80 ^1^
Horizontal meridian 2 years			
Tilt (degree, Mean ± SD)	1.66° ± 1.45°	5.06° ± 4.65°	*p* < 0.05 ^1^
Decentration (mm, Mean ± SD)	0.35 ± 0.32	0.34 ± 0.26	*p* = 0.76 ^1^
Vertical meridian 1 month			
Tilt (degree, Mean ± SD)	1.79° ± 2.19°	2.93° ± 2.55°	*p* = 0.06 ^1^
Decentration (mm, Mean ± SD)	0.39 ± 0.32	0.27 ± 0.18	*p* = 0.17 ^1^
Vertical meridian 2 years			
Tilt (degree, Mean ± SD)	1.74° ± 2.16°	3.09° ± 2.79°	*p* = 0.02 ^1^
Decentration (mm, Mean ± SD)	0.34 ± 0.33	0.27 ± 0.20	*p* = 0.45 ^1^

Abbreviations: SD—standard deviation; ^1^ U Mann-Whitney.

**Table 3:** Results of visual acuity and refractive error at two years follow-up.

Refractive Status	Four-Point Scleral Fixation (Akreos AO60)	Yamane Technique (MA60AC)	*p*-Value
BCVA (logMAR, Mean ± SD)	0.10 ± 0.15	0.09 ± 0.17	*p* > 0.05 ^1^
RE (D, Mean ± SD)	−0.08 ± 0.61	0.85 ± 0.72	*p* < 0.05 ^1^
CYL (D, Mean ± SD)	−0.47 ± 0.85	−0.52 ± 0.98	*p* > 0.05 ^1^
SE (D, Mean ± SD)	−0.44 ± 1.14	0.29 ± 1.12	*p* = 0.01 ^1^

Abbreviations: SD—standard deviation; BCVA—best-corrected visual acuity; RE—total refractive error; D—diopters; CYL—cylinder; SE—spherical equivalent; ^1^ U Mann–Whitney.

**Table 4:** Comparative characteristics of IOL tilt, decentration, and PPV in both groups at the two-years follow-up.

IOL Position	Four-Point Scleral Fixation (Akreos AO60)		*p*-Value	Yamane Technique (MA60AC)		*p*-Value
	Non-PPV (No. = 18)	PPV (No. = 7)		Non-PPV (No. = 20)	PPV (No. = 5)	
Horizontal meridian						
Tilt (degree, Mean ± SD)	1.70° ± 1.65°	1.64° ± 0.98°	*p* = 0.78 ^1^	5.33° ± 5.05°	4.42° ± 3.76°	*p* = 0.47 ^1^
Decentration (mm, Mean ± SD)	0.31 ± 0.23	0.43 ± 0.50	*p* = 0.17 ^1^	0.34 ± 0.28	0.36 ± 0.26	*p* = 0.93 ^1^
Vertical meridian						
Tilt (degree, Mean ± SD)	2.08° ± 2.42°	1.03° ± 0.95°	*p* = 0.38 ^1^	3.12° ± 2.97°	2.93° ± 1.37°	*p* = 0.52 ^1^
Decentration (mm, Mean ± SD)	0.31 ± 0.20	0.32 ± 0.41	*p* = 0.68 ^1^	0.27 ± 0.20	0.26 ± 0.21	*p* = 0.97 ^1^

Abbreviations: SD—standard deviation; no. number; non-PPV—without vitrectomy; PPV—vitrectomy. ^1^ U Mann–Whitney.

## Data Availability

The data presented in this study are available on request from the corresponding author due to privacy restrictions.
